# Flecainide toxicity—Clinical diagnosis and management of an urgent condition

**DOI:** 10.1002/ccr3.9371

**Published:** 2024-08-29

**Authors:** George Bazoukis, Polyxeni Efthymiou, Andronicos Yiannikourides, Elina Khattab, Varnavas Dimitriades, Gary Tse, Marios Pavlou, Panagiota Georgiou, Lorentzos Kapetis, Dimitrios Patestos, Michalis Tsielepis, Kimon Myrianthopoulos, Elias Papasavvas, Theodoros Christophides

**Affiliations:** ^1^ Department of Cardiology Larnaca General Hospital Larnaca Cyprus; ^2^ European University Cyprus Medical School Nicosia Cyprus; ^3^ Department of Cardiology Nicosia General Hospital Nicosia Cyprus; ^4^ Department of Cardiology, Tianjin Key Laboratory of Ionic‐Molecular Function of Cardiovascular Disease Tianjin Institute of Cardiology, Second Hospital of Tianjin Medical University Tianjin China; ^5^ School of Nursing and Health Studies Hong Kong Metropolitan University Hong Kong China; ^6^ Electrophysiology Laboratory Aretaeio Hospital Nicosia Cyprus

**Keywords:** bizarre ECG, wide QRS tachycardia, drug toxicity, flecainide toxicity

## Abstract

Clinical suspicion, clinical presentation, and electrocardiogram can help clinicians diagnose flecainide toxicity. Currently, there are no guidelines for the management of patients with flecainide toxicity. Sodium bicarbonate, lipid emulsion therapy, and extracorporeal life support have been used in this setting. Amiodarone and lidocaine can be used for the management of wide QRS complex tachycardias in hemodynamically stable patients with flecainide toxicity.

## 1 INTRODUCTION

Flecainide is a class IC antiarrhythmic drug primarily used to treat supraventricular tachycardia and atrial fibrillation in patients without structural heart disease. Flecainide acts in the fast inward sodium channel during phase 0 of the action potential.^1^ Although the therapeutic window for flecainide is narrow at 200–1000 μg/L, the reported prevalence of toxicity due to class IC antiarrhythmic is low.^2^ However, the reported mortality rate related to flecainide toxicity is as high as 22%.^2^ Diagnosis of flecainide toxicity is primarily based on clinical suspicion and electrocardiographic findings. Consequently, flecainide toxicity can be easily misdiagnosed, and no specific management guidelines currently exist. In this manuscript, we present a case of flecainide toxicity and summarize the existing data on the appropriate management of this condition.

## 2 CASE HISTORY/EXAMINATION

A 49‐year‐old woman presented to the emergency department complaining of progressive dyspnea, dizziness, and malaise. Her medical history included paroxysmal atrial fibrillation with a previous atrial fibrillation ablation procedure, surgically corrected coarctation of the aorta, transsphenoidal resection of a pituitary cyst, and chronic kidney disease. Her medication included flecainide 100 mg three times daily.

At the initial presentation, she was hemodynamically stable and she had tachypnea. Physical examination showed shortness of breath, bilateral wheezing on lung auscultation, and fast regular heart rate on cardiac auscultation.

## 3 METHODS (INVESTIGATION AND TREATMENT)

**FIGURE 1:**
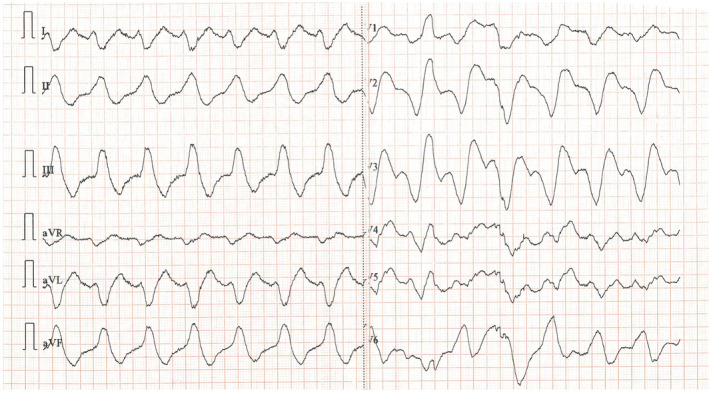
The electrocardiogram of the patient at the time of presentation. A bizzare‐looking wide QRS complex tachycardia with a rate of approximately 100 pulses per minute was revealed.

**FIGURE 2:**
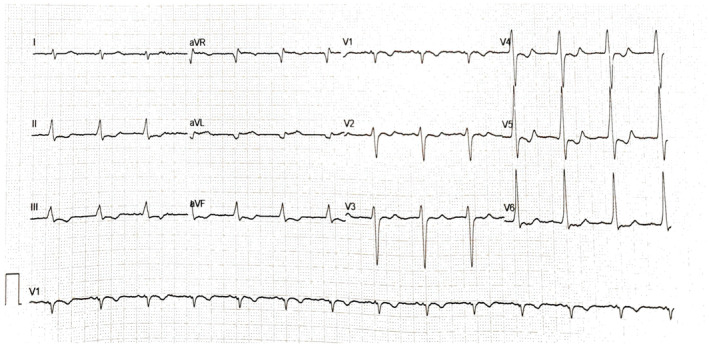
The baseline electrocardiogram 3‐months before the presentation to the emergency department. The electrocardiogram shows sinus rhythm with a rate of 83 pulses per minute. Diffuse repolarization abnormalities in the inferior and precordial leads are noted.

**FIGURE 3:**
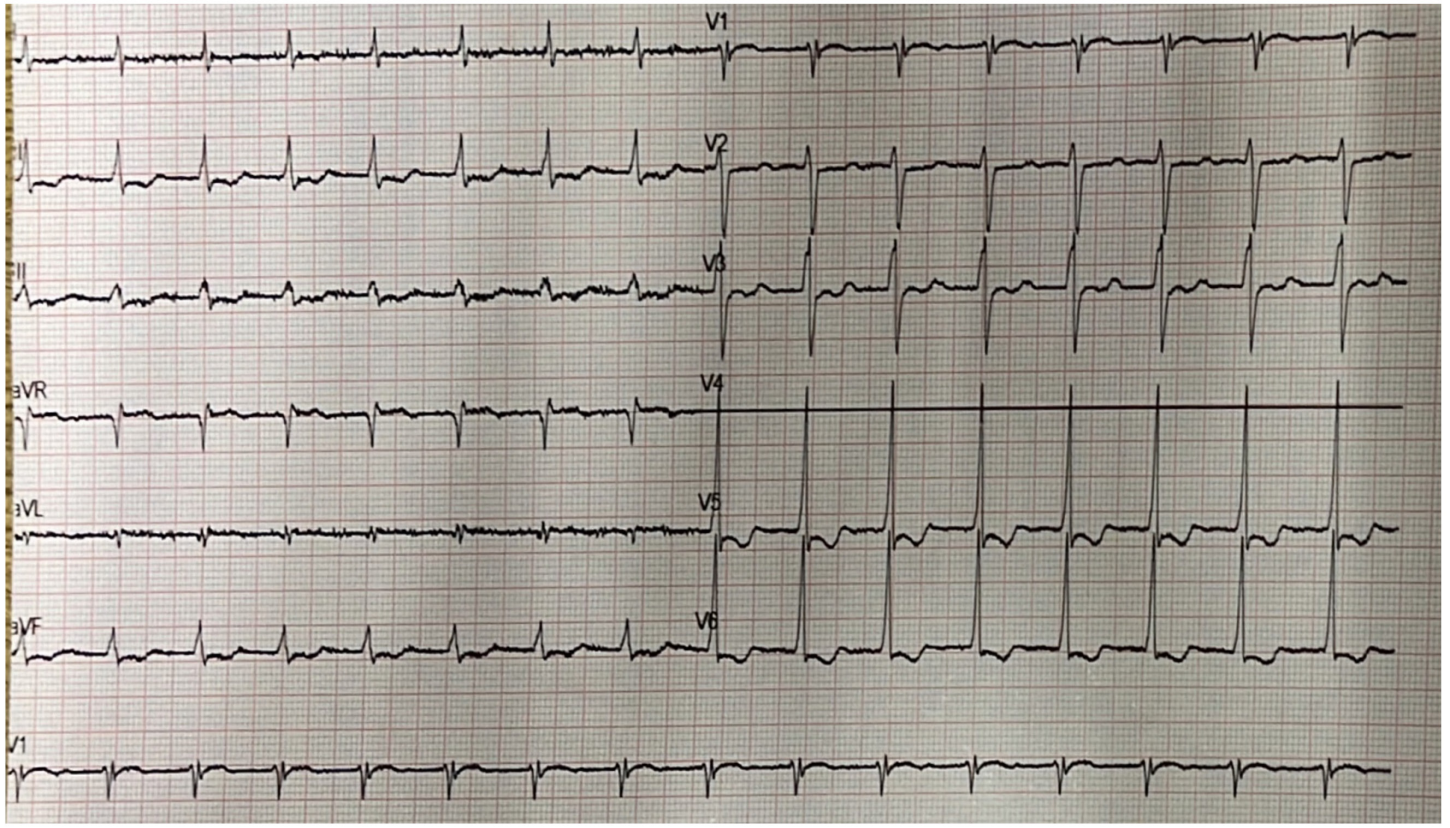
The electrocardiogram of the patient following the administration of amiodarone. A regular rhythm with a rate of 100 pulses per minute and diffuse repolarization abnormalities similar to the baseline electrocardiogram were noted. (Disconnected ECG lead V4).

Laboratory analysis revealed elevated creatinine levels (1.57 mg/dL), significant hyponatremia (sodium level 122 mmol/L) and normal potassium levels (4.54 mmol/L). Sodium levels were 126 mmol/L a few months ago. No other remarkable abnormalities were revealed. The patient had a normal urine output. The 12‐lead electrocardiogram (ECG) showed a bizarre, regular, wide QRS complex tachycardia (100 beats per minute) (Figure 1). Her baseline ECG 3 months before the presentation is demonstrated in Figure 2. Amiodarone 300 mg was administered slowly and a follow‐up ECG showed atrial fibrillation with narrow QRS complexes and a heart rate of 60 beats per minute (Figure 3). A bedside cardiac ultrasound revealed mild impairment of left ventricular systolic function and mild enlargement of the right ventricle with mildly reduced systolic function. No pericardial effusion was noted. A CT pulmonary angiogram excluded pulmonary embolism. The patient was then transferred to a tertiary hospital for coronary angiography, which excluded coronary artery disease.

## 4 CONCLUSION AND RESULTS

The clinical presentation, ECG findings, and presence of hyponatremia, combined with the exclusion of pulmonary embolism and coronary artery disease, raised the suspicion of flecainide toxicity. The patient was transferred to the intensive care unit for monitoring. Flecainide was withdrawn, and hyponatremia was corrected. During hospitalization, the patient had episodes of atrial fibrillation with a rapid ventricular response while the administration of low doses of beta blockers led to a slow heart rate (35–40/min). The implantation of a pacemaker was discussed with the patient. However, an atrial fibrillation catheter ablation procedure was decided. As we have already mentioned, the patient had ablation for persistent atrial fibrillation in the past but maintaining sinus rhythm was challenging. This was achieved using flecainide for a long time. Furthermore, a short course of amiodarone resulted in excessive bradycardia combined with QT prolongation while b‐blockers failed to control symptoms. Therefore, ablation seemed the best option. Following the ablation procedure, the patient remained in good clinical condition and the ECG showed a sinus rhythm at one‐month of follow‐up.

## 5 DISCUSSION

**FIGURE 4:**
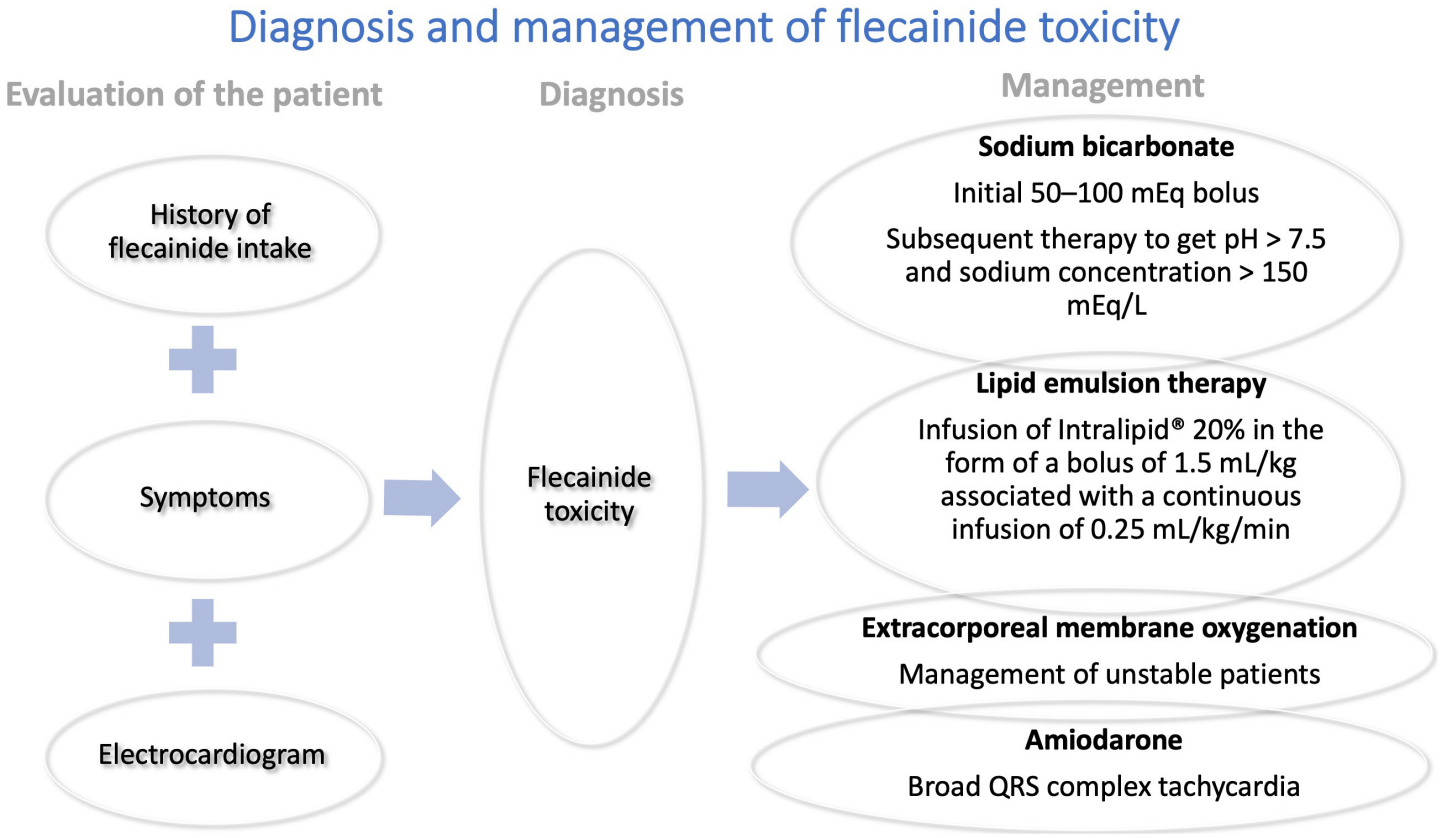
Summary of the diagnostic tools and the proposed treatment options for managing patients with flecainide toxicity.

Flecainide is used for the treatment of supraventricular and ventricular arrhythmias.^3^ Adverse effects of flecainide can be classified into cardiac and noncardiac categories. Cardiac adverse effects of flecainide include proarrhythmia, conduction abnormalities, and negative inotropic effects. Supratherapeutic levels of flecainide can cause prolongation of the PR interval, QRS duration and QT, leading to life‐threatening arrhythmias.^4^, ^5^ The most frequent non‐cardiac side effect is dizziness, followed by blurred vision and difficulty focusing.^6^ In healthy subjects, flecainide has high oral bioavailability, a high volume of distribution, and a short distribution half‐life (10 min). However, the plasma half‐life of unchanged flecainide is relatively long, averaging 13 h after a single dose and 16 h after multiple doses.^7^, ^8^ Electrolyte disturbances, such as hypokalemia and hyponatremia, as well as renal failure, can trigger toxicity. The characteristics of flecainide metabolism, combined with the absence of a specific antidote and the inability to rapidly eliminate the drug from the body, make treating overdose or toxicity challenging.^8^, ^9^ Regarding hemodialysis, it is not effective for removing flecainide but can provide a substantial removal of metabolites.^7^ The existing treatment options are summarized in Figure 4.

Early identification of flecainide toxicity is crucial for better patient outcomes. In addition to assessing flecainide intake history and symptoms, an ECG can aid in diagnosis. A review of published case reports indicates that patients with flecainide toxicity and a QRS duration ≤200 ms are more likely to show right bundle branch block (RBBB), visible P waves, and shorter QT and QTc intervals. Conversely, patients with a QRS duration >200 ms are more likely to show left bundle branch block (LBBB), loss of P waves, a northwest axis, and longer QT and QTc intervals.^10^ Interestingly, the QRS duration is related to the patient's outcomes. Specifically, deaths were reported only in patients with a QRS duration >200 ms, and the outcome of death or requirement for mechanical circulatory support was more prevalent in patients with a QRS duration >200 ms.^10^

Various strategies have been proposed for managing flecainide toxicity, though most evidence is case‐based. Sodium bicarbonate should be administered to patients with flecainide toxicity, potentially due to sodium loading and alkalinization.^10^, ^11^ Vu et al. reported that high‐dose hypertonic sodium bicarbonate is the mainstay of medical therapy for flecainide overdose and should be dosed aggressively (initial 50–100 mEq bolus with subsequent therapy to get pH >7.5 and sodium concentration >150 mEq/L).^12^ Intravenous sodium bicarbonate boluses (2 sodium bicarbonate 8.4% 50 mEq ampules) followed by an infusion (sodium chloride 0.45% with sodium bicarbonate 75 mEq) at 75 mL/h can be used.^13^

Lipid emulsion therapy is another treatment option that should be considered as a second‐line treatment.^14^ In the case of flecainide toxicity, lipid emulsion therapy could be used as an adjunct to the initial boluses of sodium bicarbonate.^10^ “Lipid Sink” theory can explain the mechanism of action. According to this theory, flecainide, a lipophilic drug, can be removed from the site of toxicity by adding large quantities of lipids to the blood.^15^ Regarding the administration, infusion of Intralipid® 20% in the form of a bolus of 1.5 mL/kg associated with a continuous infusion of 0.25 mL/kg/min has been proposed.^14^

Extracorporeal life support, combined with lipid emulsion, has been used for unstable patients with flecainide toxicity.^12^, ^16^, ^17^, ^18^, ^19^ In patients with severe cardiac dysfunction caused by flecainide intoxication, extracorporeal life support can maintain vital organ perfusion allowing time for drug metabolism, and clearance.^20^ The hemoadsorption technique based on the CytoSorb® cartridge has been used, though its efficacy remains undetermined.^18^ Managing broad QRS complex tachycardias in flecainide toxicity can be challenging. Amiodarone and lidocaine have been used in cases of toxicity, though the mechanism remains unclear.^12^, ^21^

Early diagnosis of flecainide toxicity is the cornerstone for providing the appropriate management and achieving better clinical outcomes. Clinical presentation and ECG can help clinicians to diagnose this urgent condition. The flecainide serum level may take days to result so it cannot help clinicians to diagnose toxicity early. As we have already mentioned, the therapeutic window of flecainide is narrow and ranges between 0.2 and 1.0 mcg/mL.^2^

The initial management includes the administration of sodium bicarbonate to reverse the effect of flecainide and prevent life‐threatening arrhythmias. Lipid emulsion therapy can be used as an adjunctive measure, while extracorporeal life support can be effectively used in unstable patients. Randomized clinical trials are needed to evaluate the efficacy of the case‐based proposed measures. This can lead to a standardized treatment protocol for flecainide toxicity.

## AUTHOR CONTRIBUTIONS

**George Bazoukis**: Wrote the first draft, management of the patient, approval of the final manuscript. **Polyxeni Efthymiou**: management of the patient, major revisions, approval of the final manuscript. **Andronicos Yiannikourides**: management of the patient, major revisions, approval of the final manuscript. **Elina Khattab**: management of the patient, major revisions, approval of the final manuscript. **Varnavas Dimitriades**: management of the patient, major revisions, approval of the final manuscript. **Gary Tse**: major revisions, approval of the final manuscript. **Marios Pavlou**: management of the patient, major revisions, approval of the final manuscript. **Panagiota Georgiou**: management of the patient, major revisions, approval of the final manuscript. **Lorentzos Kapetis**: management of the patient, major revisions, approval of the final manuscript. **Dimitrios Patestos**: management of the patient, major revisions, approval of the final manuscript. **Michalis Tsielepis**: management of the patient, major revisions, approval of the final manuscript. **Kimon Myrianthopoulos**: management of the patient, major revisions, approval of the final manuscript. **Elias Papasavvas**: management of the patient, major revisions, approval of the final manuscript. **Theodoros Christophides**: management of the patient, major revisions, approval of the final manuscript.

## FUNDING INFORMATION

The authors have not received any funding.

## CONFLICT OF INTEREST STATEMENT

The authors declare no conflicts of interest.

## CONSENT

Written informed consent was obtained from the patient to publish this report in accordance with the journal's patient consent policy.

## Data Availability

Data available on request from the authors.
